# Heart Rate Fragmentation: A New Approach to the Analysis of Cardiac Interbeat Interval Dynamics

**DOI:** 10.3389/fphys.2017.00255

**Published:** 2017-05-09

**Authors:** Madalena D. Costa, Roger B. Davis, Ary L. Goldberger

**Affiliations:** Beth Israel Deaconess Medical Center, Harvard Medical SchoolBoston, MA, USA

**Keywords:** aging, coronary artery disease, fragmentation index, heart rate variability, vagal tone

## Abstract

**Background:** Short-term heart rate variability (HRV) is most commonly attributed to physiologic vagal tone modulation. However, with aging and cardiovascular disease, the emergence of high short-term HRV, consistent with the breakdown of the neuroautonomic-electrophysiologic control system, may confound traditional HRV analysis. An apparent dynamical signature of such anomalous short-term HRV is frequent changes in heart rate acceleration sign, defined here as heart rate fragmentation.

**Objective:** The aims were to: (1) introduce a set of metrics designed to probe the degree of sinus rhythm fragmentation; (2) test the hypothesis that the degree of fragmentation of heartbeat time series increases with the participants' age in a group of healthy subjects; (3) test the hypothesis that the heartbeat time series from patients with advanced coronary artery disease (CAD) are more fragmented than those from healthy subjects; and (4) compare the performance of the new fragmentation metrics with standard time and frequency domain measures of short-term HRV.

**Methods:** We analyzed annotated, open-access Holter recordings (University of Rochester Holter Warehouse) from healthy subjects and patients with CAD using these newly introduced metrics of heart rate fragmentation, as well as standard time and frequency domain indices of short-term HRV, detrended fluctuation analysis and sample entropy.

**Results:** The degree of fragmentation of cardiac interbeat interval time series increased significantly as a function of age in the healthy population as well as in patients with CAD. Fragmentation was higher for the patients with CAD than the healthy subjects. Heart rate fragmentation metrics outperformed traditional short-term HRV indices, as well as two widely used nonlinear measures, sample entropy and detrended fluctuation analysis short-term exponent, in distinguishing healthy subjects and patients with CAD. The same level of discrimination was obtained from the analysis of normal-to-normal sinus (NN) and cardiac interbeat interval (RR) time series.

**Conclusion:** The fragmentation framework and accompanying metrics introduced here constitute a new way of assessing short-term HRV under free-running conditions, one which appears to overcome salient limitations of traditional HRV analysis. Fragmentation of sinus rhythm cadence may provide new dynamical biomarkers for probing the integrity of the neuroautonomic-electrophysiologic network controlling the heartbeat in health and disease.

## Introduction

**Figure 1:**
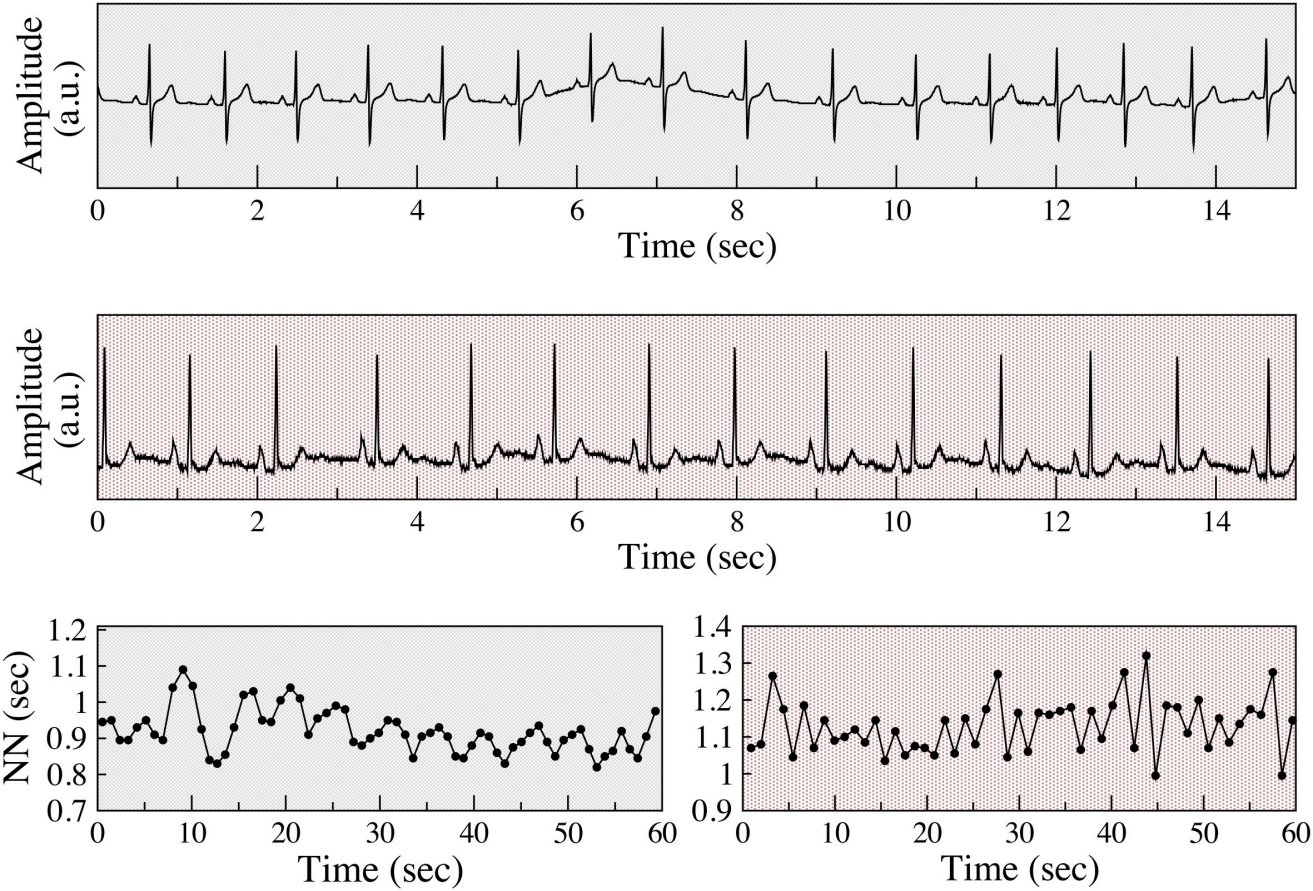
**Examples of respiratory sinus arrhythmia and anomalous sinus rhythm**. Electrocardiograms (Holter lead) from a healthy subject **(top)** and a patient with coronary artery disease (CAD) **(middle)**, both from the present study. Normal-to-normal (NN) sinus interval time series from the healthy subject **(bottom left)** and of the patient with CAD **(bottom right)**. The fluctuation patterns of the former time series are characteristic of phasic (respiratory) sinus arrhythmia, while that of the latter are indicative of an abnormal non-phasic sinus arrhythmia. To assist in visual comparisons, pale gray backgrounds are used for data from the healthy subject and light red for data from the patient with CAD, respectively. ECG voltage is given in arbitrary units (a.u).

Heart rate variability (HRV) in healthy subjects, particularly over short time scales, is primarily attributable to fluctuations in vagal tone. The most recognizable manifestation of this parasympathetic influence is the oscillatory RR interval pattern (Figure 1) termed respiratory sinus arrhythmia (RSA) that results from the coupling between breathing and heart rate (Angelone and Coulter, 1964; Hirsch and Bishop, 1981; HRV, 1996; Stauss, 2003). However, beat-to-beat changes in the heart rate of *healthy* subjects not synchronized with respiration are also vagally mediated (Angelone and Coulter, 1964; Hirsch and Bishop, 1981). Therefore, a central interpretative framework underlying contemporary HRV analyses is one in which the degree of short-term variability of normal-to-normal (NN) sinus beats is used as a dynamical biomarker of cardiac vagal tone modulation (HRV, 1996; Billman, 2011).

This topic is of particular importance because parasympathetic regulation of sinus rhythm decreases with aging and organic heart disease (HRV, 1996; Kuo et al., 1999; Thayer et al., 2010). However, paradoxically, for some subjects in these high risk groups the amount of short-term variability actually increases (Figure 1). An apparent difference in the time series of vagally and non-vagally mediated HRV dynamics is their degree of smoothness, or conversely, their degree of fragmentation. Vagal tone modulation changes the heart rate in a progressive way. For example, with RSA, heart rate gradually increases and decreases with inspiration and expiration, respectively. When the coupling between heart rate and respiration is not as apparent, but the changes in heart rate are still driven by vagal tone modulation, the changes in heart rate are also gradual. In contrast, non-vagally mediated, short-term heart rate variability has a distinct dynamical signature, namely more frequent changes in heart rate acceleration sign (Figure 1). In the “extreme” case of sinus alternans, the sign of heart rate acceleration changes every beat (Lewis, 1920; Geiger and Goerner, 1945; Friedman, 1956; Binkley et al., 1995). The presence of these abnormal variants of sinus rhythm limits the utility of traditional HRV analysis, since an increase in the overall amount of short-term variability can no longer be solely attributed to enhanced vagal tone modulation.

Stein et al. (Domitrovich and Stein, 2002; Stein, 2002), coined the term “erratic sinus rhythm” to refer to prominent but apparently random variations in sinus cadence not attributable to vagal tone modulation and proposed a semi-quantitative approach to help identify them (Stein et al., 2005, 2008). However, despite their association with increased cardiovascular risk and sick sinus syndrome (Bergfeldt and Haga, 2003), erratic sinus rhythm, sinus alternans, and their variants, have received scant clinical attention and the underlying mechanisms remain obscure.

As shown in Figure 1, the distinctions between the different classes of sinus arrhythmia may be difficult or impossible to discern from standard ECG recordings. The graphs of the NN interval time series and other representations of the data, such as Poincaré plots and Fourier spectra (not shown) may reveal clear differences in the structure of the fluctuations between physiologic and anomalous variability. However, in many cases, the differences are difficult to identify and especially to quantify.

These considerations led to the development of a novel approach to the analysis of short-term heart rate variability, termed *heart rate fragmentation*, accompanied by a set of simple-to-implement statistical metrics. A framework for the proposed approach is the concept that adaptive control of the heartbeat, particularly on short time scales, requires a hierarchy of interacting networks comprising neuroautonomic (especially the parasympathetic) and electrophysiologic components (sinus node pacemaker cells and their connections to the atrial syncytium). The integrity of these networks allows for their correlated function, evinced in part by the smoothness (fluency) of the output. At the same time, their functionality provides for sufficiently rapid (short-term or high frequency) responsiveness to physiologic stresses, while protecting against excessive volatility on a beat-to-beat basis.

A corollary concept is that network dysfunction, in general, and of the heart rate control system, in particular, is more likely to occur as the components of the network and their physiologic coupling start to break down. This degradative process should lead to increasing degrees of fragmentation. A key aspect of the fragmentation paradigm is that dysfunction or actual breakdown of one or more system components allows for the emergence of high frequency fluctuations that compete with or even exceed the shortest-term modulatory responsiveness of the vagal system. Therefore, a marker of this fragmentation on the surface ECG should be abrupt changes in the sign of heart rate acceleration, which may be periodic (as with classic sinus node alternans) or more random appearing (as with what has been termed “erratic sinus rhythm”). Such markers of fragmentation may be useful as correlates of cardiovascular aging and/or underlying organic heart disease.

Accordingly, we developed a set of fragmentation indices (see Methods) and applied them to beat-annotated, well-characterized 24-h Holter monitor recordings obtained from two very distinct clinical groups: healthy subjects and those with coronary artery disease (CAD). We analyzed three different time periods: the full day, putative awake and sleep periods. The primary hypotheses were that: (1) heart rate fragmentation would be higher in healthy old subjects than in younger ones for all three time periods; and (2) heartbeat time series from patients with CAD would be more fragmented than those from healthy subjects. We also tested whether the fragmentation indices would outperform standard time and frequency domain measures, as well as nonlinear measures of short-term HRV in classifying heart rate time series from healthy subjects *vs*. those from patients with CAD.

## Methods

### Databases

We employed two long-term (~24-h) ECG ambulatory databases from the Intercity Digital Electrocardiogram Alliance (IDEAL) study. The recordings are made available via the University of Rochester Telemetric and Holter ECG Warehouse (THEW) archives (http://thew-project.org/databases.htm).

1. Healthy Subjects Database (THEW identification: E-HOL-03-0202-003)
  The database comprises 24-h Holter recordings from 202 ostensibly healthy subjects (102 males). Subjects were not pregnant and had (1) no overt cardiovascular disease or history of cardiovascular disorders; (2) no reported medications, (3) a normal physical examination, (4) a 12-lead ECG showing sinus rhythm with normal waveforms (or a normal echocardiogram and normal ECG exercise testing in the presence of any questionable findings ECG changes). The ECG signals were recorded at a sample frequency of 200 Hz. Automated beat annotations were manually reviewed and adjudicated. We excluded 45 subjects with more than 1% non-sinus beats, 37 younger than 25 years old, ten with body mass index >30 Kg/m^2^ and one with <12 h of data. Overall, we analyzed data from 109 healthy adult subjects (60 male), age (median, 25–75th percentiles) 40, 33–49 years.
2. Coronary Artery Disease Subjects Database (THEW identification E-HOL-03-0271-002)
  This database comprises 24-h Holter recordings from 271 patients (223 males). Subjects had an abnormal coronary angiogram (at least one vessel with luminal narrowing >75%) and either exercise-induced ischemia or a documented previous myocardial infarction. Exclusion criteria included a history of coronary artery bypass surgery or major co-morbidity. Patients were clinically stable and in sinus rhythm at the time of the enrollment. For our analysis, we also excluded 11 subjects whose Holter recordings contained ≥20% non-sinus beats and 4 with less than 12 h of data. Overall, we analyzed 256 subjects (208 male), age (median, 25–75th percentiles): 60; 51–67 years; left ventricular ejection fraction 56.5, 50–66%.

Putative waking and sleeping periods were estimated as the six consecutive hours of highest and lowest heart rates, respectively. These periods were calculated from the NN interval time series using a 6-h moving average window, shifted 15 min at a time.

### HRV analysis: heart rate fragmentation indices

From the ECG of each subject, the time series of the NN intervals, {*NN*_*i*_} = {*t*_*N*_*i*__ − *t*_*N*_*i*−1__}, where *t*_*N*_*i*__ represents the time of occurrence of the *i*^*th*^ normal sinus beat, and the time series of the differences between consecutive NN intervals (increments), {Δ*NN*_*i*_} = {*NN*_*i*_ − *NN*_*i*−1_}, were derived.

The following four fragmentation indices were then computed from these time series:

- The percentage of zero-crossing points in the increment time series, or equivalently, the percentage of inflection points (PIP) in the NN interval time series. (A *t*_*N*_*i*__ represents an inflection point if Δ*NN*_*i*_ × Δ*NN*_*i*+1_ ≤ 0, that is, if *t*_*N*_*i*__ is an instant of inversion of heart rate acceleration sign or of change to or from zero.)
- The inverse of the average length of the acceleration/deceleration segments (IALS). An acceleration, deceleration segment is a sequence of NN intervals between consecutive inflection points for which the difference between two NN intervals is <0 and >0, respectively. The length of a segment is the number of NN intervals in that segment.
- The complement of the percentage of NN intervals in acceleration and deceleration segments with three or more NN intervals. This quantity is termed the percentage of short segments (PSS).
- The percentage of NN intervals in alternation segments. An alternation segment is a sequence of at least four NN intervals, for which heart rate acceleration changes sign every beat. Such sequences follow an “ABAB” pattern, where “A” and “B” represent increments of opposite sign. This quantity is abbreviated, PAS.

By definition, the more fragmented a time series is, the higher the PIP, IALS, PSS, and PAS indices will be. We note that PAS quantifies the amount of a particular sub-type of fragmentation (alternation). A time series may be highly fragmented and have a small amount of alternation. However, all time series with large amount of alternation are highly fragmented.

Given that the presence of non-sinus beats will increase fragmentation, we excluded segments encompassing non-sinus beats that started and ended at the inflection points preceding and following these non-sinus beats, respectively.

Finally, to assess the importance of beat annotation on fragmentation analyses, we also examined the full RR time series that include normal sinus beats as well as any supraventricular and ventricular ectopic beats.

### HRV analysis: standard measures

Standard techniques of HRV analysis are grouped into time and frequency (spectral) domain methods (HRV, 1996). A subset of the former, intended to quantify short-term variability, is based on the difference between consecutive normal-to-normal intervals (ΔNN, also termed NN increments); the latter on the spectral power of the NN intervals.

The following four traditional time and frequency HRV measures of short-term fluctuations were computed using open-source software (Goldberger et al., 2000) available at the PhysioNet website (www.physionet.org):

- Time domain
  – pNNx measures: the percentage of ΔNN > *x* ms. Here, we used *x* = 20 and 50 ms (Mietus et al., 2002).
  – rMSSD (“root mean square of successive differences”): square root of the mean of the squares of ΔNN intervals.
  – SDSD (“standard deviation of successive differences”): standard deviation of the ΔNN time series.
- Frequency domain
  – HF (“high frequency”): spectral power of the NN interval time series between 0.15 and 0.4 Hz.

These sets of time and frequency domain measures are widely interpreted to represent cardiac vagal tone modulation (HRV, 1996; Billman, 2011). By comparison, longer time scale fluctuations, are attributable to both sympathetic and parasympathetic influences (HRV, 1996; Thayer et al., 2010; Billman, 2013) and were, therefore, not considered here.

### HRV analysis: non-linear dynamical indices

The following two widely used nonlinear short-term dynamical indices were computed:

- Short-term detrended fluctuation analysis (DFA) exponent, α_1_. This measure (Peng et al., 1995) quantifies the correlations properties of a time series. The method is based on the assessment of the slope of the linear regression line of the log-log graph of *F*(*n*) *vs*. *n*. The function *F*(*n*) is the root-mean-square fluctuation of the integrated and detrended data, computed using windows of length *n*. For the analysis of heart rate time series, two indices, α_1_ and α_2_, quantifying short and long-term behavior, respectively, have been proposed. Here, we focus on α_1_, which encompasses scales ranging from 4 to 11 beats, inclusively (Pikkujamsa et al., 1999). The correlation properties of time series with α ≃ 1.5 are similar to those of Brownian noise. In contrast, time series with α < 0.5 are anti-correlated. The former are smoother than the latter.
- Sample entropy (SampEn). This measure (Richman and Moorman, 2000) quantifies the degree of irregularity of a signal. A higher SampEn value implies a more irregular, less predictable signal. Sample entropy is the negative of the natural logarithm of the conditional probability that the (*m* + 1)^*th*^ components of two distinct segments match (||*x*_*i*+*m*_ − *x*_*j*+*m*_|| < *r*) within the tolerance *r*, given that the first *m* components match within the same tolerance (||*x*_*i*+*l*_ − *x*_*j*+*l*_|| < *r*, for 0 ≤ *l* ≤ *m* − 1).

### Statistical analysis

Spearman's rank and Pearson's product-moment correlation coefficients were used to quantify the dependence of: (i) the four novel indices of heart rate fragmentation, (ii) the traditional measures of short-term HRV, and iii) the two non-linear dynamical indices, short-term DFA exponent α_1_ and SampEn, with the participants' age, using the THEW Healthy Subject Database. Statistical significance was set at a *p*-value <0.05.

Logistic regression analysis methods were used to assess the relationships between presence of CAD and traditional, nonlinear and fragmentation indices in unadjusted models and models adjusted for age and gender. To facilitate comparisons among various HRV measures, we report normalized odds ratios (i.e., the odds ratio for a one standard deviation change in the measure).

The area under the receiver operating characteristic (AUC) curve was used to assess the goodness of fit of each model. The likelihood-ratio test was used to compare the goodness of fit of two nested models. All analyses were performed using raw measures except in the case of skewed variables whose logarithmic or quadratic transformation improved the models' goodness of fit. This improvement was only noted in the case of 24-h and daytime HF, 24-h and daytime SDSD and nighttime α_1_.

## Results

### Changes in heart rate dynamics with the participants' age in the healthy population

**Figure 2:**
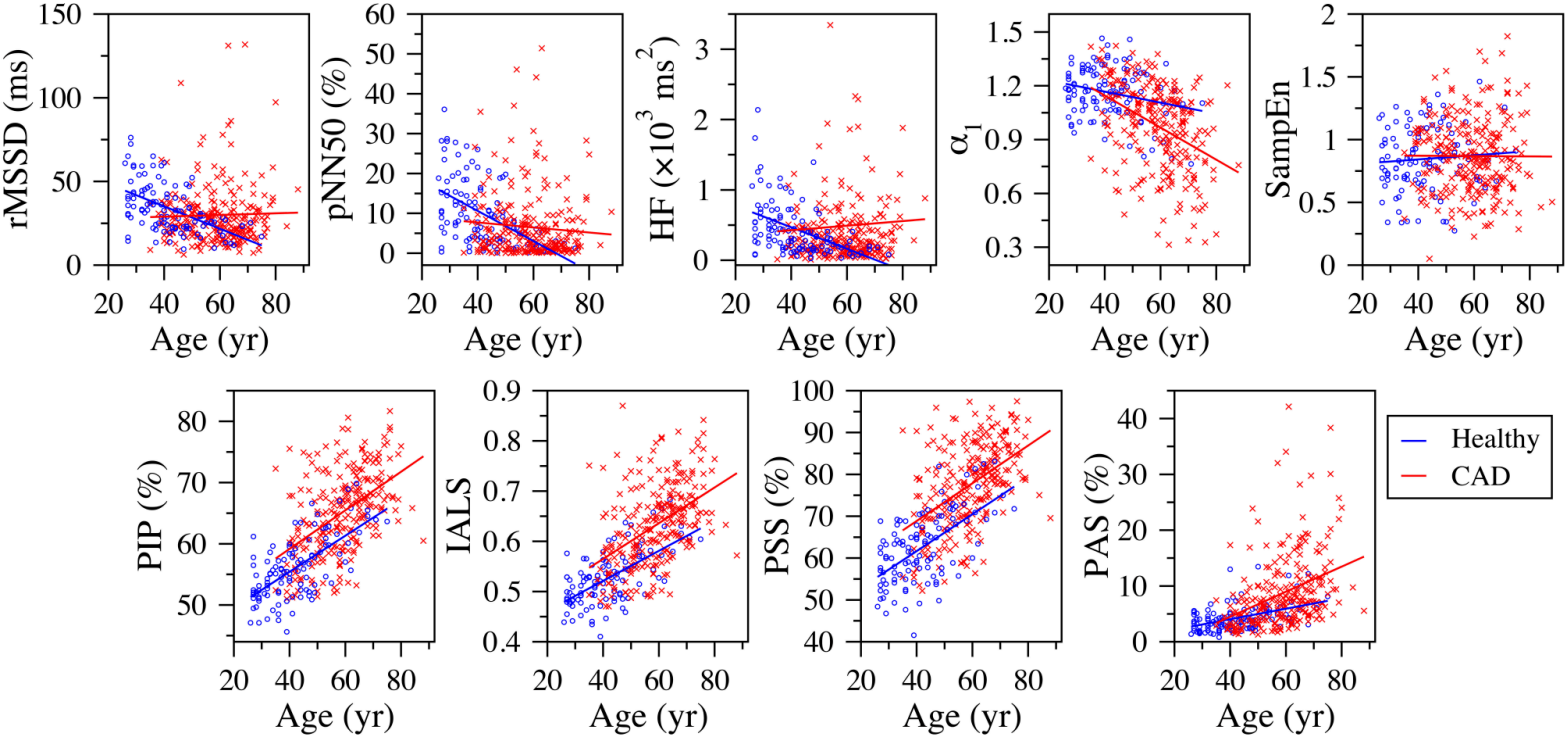
**Scatter plots of the traditional heart rate variability (rMSSD, pNN50, and HF), nonlinear (α_**1**_ and SampEn) and fragmentation (PIP, IALS, PPS, and PAS) indices vs. the participants' age for the group of healthy subjects and patients with coronary artery disease (CAD), derived from the analysis of the full (~ 24-h) period**. The solid lines are the linear regression lines. rMSSD, root mean square of the successive differences; pNN50, percentage of differences between successive NN intervals above 50 ms; HF, high frequency spectral power; α_1_, detrended fluctuation analysis short-term exponent; SampEn, sample entropy; PIP, percentage of inflection points; IALS, inverse of the average length of the acceleration/deceleration segments; PPS, percentage of NN intervals in short segments; PAS, percentage of NN intervals in alternation segments.

All four fragmentation indices significantly increased with the participants' age, for all three time periods, using either NN or RR interval time series (Table 1, Figure 2).

**Table 1:** **Spearman rank and standardized Pearson product-moment correlation coefficients for the relationships between traditional short-term HRV, nonlinear and fragmentation indices with cross-sectional age for the group of healthy subjects**.

	**Variables**	**Spearman**						**Pearson**					
		**24-h**		**Day**		**Night**		**24-h**		**Day**		**Night**	
		***r*_*s*_**	***p*-value**	***r*_*s*_**	***p* value**	***r*_*s*_**	***p*-value**	***r***	***p*-value**	***r***	***p*-value**	***r***	***p*-value**
Fragmentaion (using NN)	PIP	0.614	<0.0001	0.582	<0.0001	0.418	<0.0001	0.659	<0.0001	0.624	<0.0001	0.469	<0.0001
	IASL	0.577	<0.0001	0.555	<0.0001	0.343	0.0003	0.629	<0.0001	0.603	<0.0001	0.392	<0.0001
	PSS	0.568	<0.0001	0.595	<0.0001	0.193	0.0445	0.597	<0.0001	0.637	<0.0001	0.216	0.0240
	PAS	0.430	<0.0001	0.326	0.0006	0.432	<0.0001	0.450	<0.0001	0.324	0.0006	0.416	<0.0001
Fragmentation (using RR)	PIP	0.612	<0.0001	0.583	<0.0001	0.421	<0.0001	0.659	<0.0001	0.623	<0.0001	0.470	<0.0001
	IASL	0.576	<0.0001	0.555	<0.0001	0.346	0.0002	0.628	<0.0001	0.603	<0.0001	0.396	<0.0001
	PSS	0.568	<0.0001	0.596	<0.0001	0.197	0.0399	0.597	<0.0001	0.636	<0.0001	0.218	0.0230
	PAS	0.434	<0.0001	0.328	0.0005	0.437	<0.0001	0.450	<0.0001	0.322	0.0007	0.422	<0.0001
Traditional short-termHRV	rMSSD	−0.529	<0.0001	−0.468	<0.0001	−0.538	<0.0001	−0.524	<0.0001	−0.449	<0.0001	−0.537	<0.0001
	pNN20	−0.487	<0.0001	−0.439	<0.0001	−0.505	<0.0001	−0.496	<0.0001	−0.434	<0.0001	−0.558	<0.0001
	pNN50	−0.536	<0.0001	−0.491	<0.0001	−0.529	<0.0001	−0.518	<0.0001	−0.444	<0.0001	−0.516	<0.0001
	SDSD	−0.530	<0.0001	−0.450	<0.0001	−0.476	<0.0001	−0.523	<0.0001	−0.434	<0.0001	−0.475	<0.0001
	HF	−0.520	<0.0001	−0.439	<0.0001	−0.568	<0.0001	−0.466	<0.0001	−0.404	<0.0001	−0.481	<0.0001
Non-linear	α_1_	−0.148	0.124	−0.118	0.224	0.418	<0.0001	−0.246	0.0101	−0.211	0.0277	0.384	<0.0001
	SampEn	0.071	0.462	−0.251	0.0086	−0.225	0.0186	0.075	0.440	−0.271	0.0044	−0.265	0.0054

*PIP, percentage of inflection points; IALS, inverse of the average length of the acceleration/deceleration segments; PPS, percentage of NN intervals in short segments; PAS, percentage of NN intervals in alternation segments. rMSSD, root mean square of the successive differences; pNN20 and pNN50, percentage of differences between successive NN intervals above 20 and 50 ms, respectively; SDSD, standard deviation of successive differences; HF, high frequency spectral power; α_1_, detrended fluctuation analysis short-term exponent; SampEn, sample entropy*.

Traditional short-term HRV indices significantly decreased with the participants' age, for all three time periods.

The fractal α_1_ exponent significantly increased with the participants' age during putative sleep time. For the other time periods, linear correlation analysis indicated an inverse relationship. However, in these cases, the Spearman coefficients were not significant.

Sample entropy significantly decreased with the participants' age during the putative wake and sleep periods. However, analyses of the 24-h period did not reveal any significant association between the two variables.

The percentage of supraventricular and ventricular premature beats significantly increased with the participants' age (Spearman *r*_*s*_ = 0.27, p = 0.004 and *r*_*s*_ = 0.29, p = 0.002, respectively).

### Changes in heart rate dynamics with coronary artery disease

The values (median, 25 and 75th percentiles) of the new fragmentation indices for the groups of healthy subjects and patients with CAD, as well as of the traditional HRV and nonlinear indices, are presented in Table 2.

**Table 2:** **Measures of heart rate variability in healthy subjects and those with coronary artery disease (CAD)**.

		**Healthy**			**CAD**		
		**24-h**	**Day**	**Night**	**24-h**	**Day**	**Night**
	**Variable**	**Median, 25-75th**	**Median, 25-75th**	**Median, 25-75th**	**Median, 25-75th**	**Median, 25-75th**	**Median, 25-75th**
Fragmentation (using NN)	PIP (%)	55.4, 52.1–59.1	56.5, 52.1–61.5	54.0, 51.4–58.3	65.4, 60.1–69.4	67.0, 61.0–72.7	61.9, 57.7–67.8
	IASL	0.52, 0.49–0.56	0.53, 0.48–0.58	0.51, 0.49–0.56	0.63, 0.57–0.68	0.65, 0.58–0.72	0.59, 0.55–0.66
	PSS (%)	62.5, 56.4–68.8	61.1, 53.6–68.9	66.4, 59.5–73.7	78.1, 70.4–84.9	79.4, 69.5–87.8	77.3, 69.9–83.9
	PAS (%)	3.79, 2.66–5.10	4.36, 3.24–6.52	1.85, 1.17–3.49	7.04, 4.27–10.8	7.89, 4.80–13.2	4.29, 2.41–7.24
Fragmentation (using RR)	PIP (%)	55.5, 52.2–59.2	56.8, 52.1–61.4	54.1, 51.4–58.4	65.7, 60.3–69.6	67.2, 61.1–72.8	62.1, 57.8–68.0
	IASL	0.52, 0.49–0.56	0.53, 0.48–0.58	0.51, 0.49–0.56	0.63, 0.57–0.68	0.65, 0.58–0.72	0.60, 0.55–0.66
	PSS (%)	62.5, 56.4–68.6	61.0, 53.3–68.7	66.4, 59.5–73.7	78.1, 70.1–84.5	79.1, 69.4–87.6	77.0, 69.7–83.7
	PAS (%)	3.84, 2.71–5.10	4.36, 3.24–6.55	1.84, 1.20–3.68	7.17, 4.43–11.4	8.24, 4.86–13.7	4.48, 2.54–7.48
Traditional short-termHRV	rMSSD (ms)	30.7, 23.1–42.5	22.5, 16.5–31.7	42.1, 29.5–62.0	25.6, 18.4–34.7	20.0, 15.0–28.3	28.8, 21.5–43.6
	pNN20 (%)	32.0, 21.5–44.6	22.4, 11.8–36.1	52.6, 33.0–64.1	25.2, 15.0–37.2	17.7, 8.46–30.4	35.7, 19.9–51.9
	pNN50 (%)	7.65, 3.04–15.2	2.81, 1.03–8.13	16.0, 5.80–29.6	3.48, 1.07–8.44	1.60, 0.45–4.99	4.90, 1.40–15.6
	SDSD (ms)	31.4, 23.1–42.6	22.7, 16.2–31.8	39.9, 26.3–58.4	26.4, 18.9–36.2	20.2, 15.7–29.0	28.0, 20.6–39.7
	HF (msec^2^)	322, 173–603	159, 85.9–330	608, 283–1452	255, 128–492	140, 67.7–293	311, 156–672
Non-linear	α_1_	1.17, 1.07–1.27	1.44, 1.32–1.52	1.21, 1.04–1.34	1.02, 0.81–1.15	1.23, 1.02– 1.38	1.23, 1.02–1.37
	SampEn	0.84, 0.68–1.05	0.84, 0.63–1.07	1.32, 1.12–1.52	0.86, 0.64–1.05	0.85, 0.63– 1.02	1.21, 1.03–1.42

*Values are reported as median, 25–75th percentiles. PIP, percentage of inflection points; IALS, inverse of the average length of the acceleration/deceleration segments; PPS, percentage of NN intervals in short segments; PAS, percentage of NN intervals in alternation segments. rMSSD, root mean square of the successive differences; pNN20 and pNN50, percentage of differences between successive NN intervals above 20 ms and 50 ms, respectively; SDSD, standard deviation of successive differences; HF, high frequency spectral power; α_1_, detrended fluctuation analysis short-term exponent; SampEn, sample entropy*.

All fragmentation indices significantly (*p* < 0.0001) increased with the participants' age for all time periods in the group of patients with CAD, regardless of using NN or RR time series (Figure 2). The Pearson correlation coefficients varied between 0.250 and 0.529 for the NN time series and between 0.246 and 0.531 for the RR time series. The correlations for the 24-h and putative awake periods were relatively stronger than those for the sleep period.

Out of the 15 relationships tested, between each of the five traditional HRV measures and the participants' age, for each of the three time periods, only two were statistically significant. We found that pNN20 and pNN50 significantly decreased with the participants' age during the putative sleep period. Of the nonlinear indices, only DFA α_1_ showed a significant association with participants' age. In this group, α_1_ significantly decreased with the participants' age for all time periods.

A 1-year increase in age was associated with an increase of 14% in the odds of having CAD (odds ratio = 1.14, 95% confidence interval: 1.11–1.17, *p* < 0.0001). The AUC for the model with age as the only covariate was 0.853. Male sex carried a 3.54 fold increase in the odds of CAD (odds ratio = 3.54, 95% confidence interval: 2.17–5.78, *p* < 0.0001). The AUC for the null model with age and gender as the sole independent variables was 0.882.

#### Unadjusted analyses

In unadjusted analyses (Table 3), higher fragmentation indices were significantly associated with presence of CAD, for all time periods, using both NN and RR interval time series. Depending of the specific index and time period considered, a one-standard deviation increase in any of the fragmentation indices was associated with a 2.84–7.34-fold increase in the odds of CAD.

**Table 3:** **Logistic regression analysis and area under the ROC curve for unadjusted models of CAD**.

		**24-h**			**Day**			**Night**		
	**CAD**	**OR_n_**	**95% CI**	**AUC**	**OR_n_**	**95% CI**	**AUC**	**OR_n_**	**95% CI**	**AUC**
Fragmentation (using NN)	PIP	5.86	3.97–8.66	0.850	4.28	3.06–5.99	0.822	4.50	3.11–6.52	0.806
	IASL	6.72	4.40–10.3	0.854	4.66	3.25–6.68	0.823	4.66	3.16–6.88	0.804
	PSS	6.16	4.16–9.11	0.863	4.69	3.33–6.59	0.840	2.88	2.16–3.83	0.755
	PAS	6.60	3.58–11.4	0.762	4.14	2.57–6.69	0.727	6.42	3.21–12.8	0.750
Fragmentation (using RR)	PIP	6.06	4.08–9.00	0.854	4.42	3.15–6.12	0.826	4.61	3.17–6.69	0.809
	IASL	6.77	4.42–10.4	0.856	4.72	3.29–6.77	0.825	4.66	3.16–6.86	0.805
	PSS	6.12	4.14–9.04	0.862	4.69	3.33–9.59	0.840	2.84	2.14–3.77	0.752
	PAS	7.34	4.02–13.4	0.774	4.54	2.79–7.38	0.739	7.13	3.55–14.3	0.760
Traditional short-termHRV	rMSSD	0.835	0.673–1.04	0.616	0.957	0.769–1.19	0.556	0.735	0.589– 0.917	0.653
	pNN20	0.693	0.551–0.871	0.610	0.792	0.634–0.989	0.574	0.565	0.443– 0.720	0.656
	pNN50	0.696	0.560–0.867	0.648	0.826	0.668–1.02	0.602	0.597	0.477– 0.746	0.664
	SDSD	0.827	0.663–1.03	0.598	0.901	0.722–1.12	0.549	0.778	0.626– 0.967	0.641
	HF	0.821	0.654–1.03	0.565	0.909	0.726–1.14	0.540	0.872	0.704– 1.08	0.653
Non-linear	α_1_	0.332	0.237–0.465	0.734	0.224	0.148–0.341	0.776	1.03	0.820– 1.28	0.521
	SampEn	1.08	0.858–1.35	0.513	0.997	0.797–1.25	0.508	0.737	0.586– 0.927	0.591

*Values presented are normalized odds ratio (OR_n_), 95% confidence intervals (95% CI) and area under the receiver operating characteristic curve (AUC). PIP, percentage of inflection points; IALS, inverse of the average length of the acceleration/deceleration segments; PPS, percentage of NN intervals in short segments; PAS, percentage of NN intervals in alternation segments. rMSSD, root mean square of the successive differences; pNN20 and pNN50, percentage of differences between successive NN intervals above 20 ms and 50 ms, respectively; SDSD, standard deviation of successive differences; HF, high frequency spectral power; α_1_, detrended fluctuation analysis short-term exponent; SampEn, sample entropy. The analysis was performed using raw measures except in the case of 24-h and daytime HF, 24-h and daytime SDSD and nighttime α_1_ variables, for which the models with the transformed variables (log in the case of HF and SDSD, and square in the case of α_1_) fitted the data better than those with the raw variables*.

In comparison, the traditional short-term time and frequency domain HRV measures were inversely associated with presence of CAD for all time periods. However, only a subset of these measures, rMSSD and SDSD during sleep time, pNN50 during sleep and the 24-h period and pNN20 for all time periods, were significantly associated with CAD in unadjusted models. Of note, these models consistently performed worse than those with the fragmentation indices. For example, for pNN20, the best performing of the HRV measures, a one-standard deviation increase in the value of this variable was only associated with a 26, 77, and 44% increase in the odds of CAD, for the awake, sleep and 24-h periods, respectively.

Lower values of α_1_, for the awake and 24-h periods, and of SampEn for the sleep period were also significantly associated with presence of CAD. Overall, α_1_ was a stronger correlate of CAD than traditional HRV measures, but not as strong as the fragmentation indices. SampEn, even for the sleep period, was among the weakest correlates of CAD.

**Figure 3:**
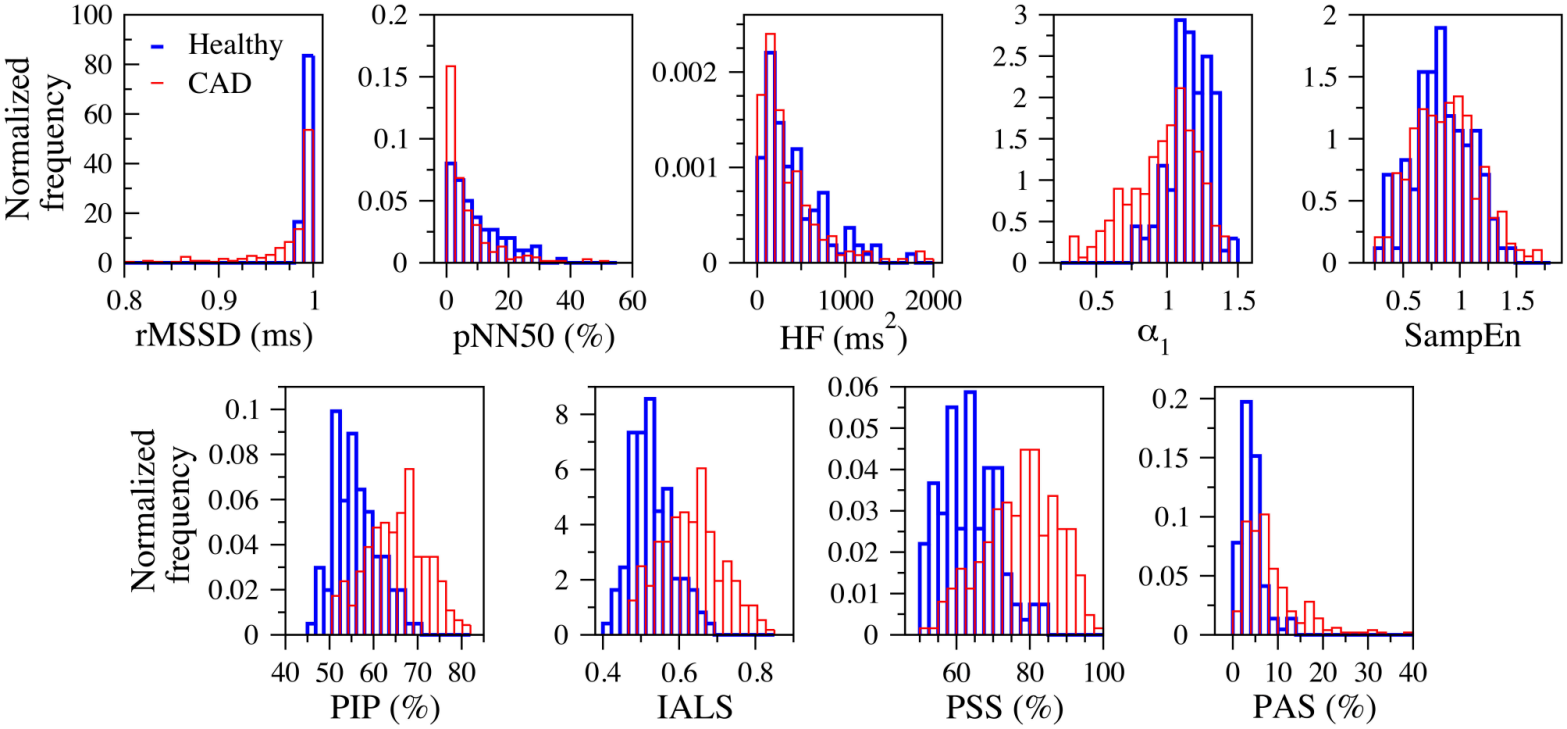
**Normalized histograms of the traditional heart rate variability (rMSSD, pNN50, and HF), nonlinear (α_**1**_ and SampEn) and fragmentation (PIP, IALS, PPS, and PAS) indices for the groups of healthy subjects (blue) and patients with coronary artery disease (red), for the 24-h period**. rMSSD, root mean square of the successive differences; pNN50, percentage of differences between successive NN intervals above 50 ms; HF, high frequency spectral power; α_1_, detrended fluctuation analysis short-term exponent; SampEn, sample entropy; PIP, percentage of inflection points; IALS, inverse of the average length of the acceleration/deceleration segments; PPS, percentage of NN intervals in short segments; PAS, percentage of NN intervals in alternation segments.

Figure 3 shows the normalized histograms of the traditional heart rate variability (rMSSD, pNN50, and HF), nonlinear (α_1_ and SampEn) and fragmentation (PIP, IALS, PPS, and PAS) indices for the groups of healthy subjects and patients with coronary artery disease, for the 24-h period.

#### Adjusted analyses

Fragmentation indices remained positively associated with CAD in models adjusted for age and sex (Table 4). Furthermore, the models with any of these indices fitted the data better than the ones with only age and sex, for all time periods, regardless of whether NN or RR time series were used.

**Table 4:** **Logistic regression analysis and AUC for models of CAD adjusted for age and sex**.

		**24-h**				**Day**				**Night**			
	**CAD**	**OR_n_**	**95% CI**	**AUC**	***p***	**OR_n_**	**95% CI**	**AUC**	***p***	**OR_n_**	**95% CI**	**AUC**	***p***
Fragmentation (NN)	PIP	2.99	1.84–4.85	0.904	8.2E-7	2.20	1.44–3.36	0.896	1.3E-4	2.64	1.70–4.10	0.901	2.0E-6
	AISL	3.31	1.97–5.55	0.906	2.0E-7	2.29	1.48–3.55	0.897	5.6E-5	2.83	1.80–4.47	0.903	5.7E-7
	PSS	3.68	2.26–5.98	0.912	2.2E-9	2.48	1.62–3.80	0.900	7.7E-6	2.51	1.74–3.63	0.905	1.0E-7
	PAS	2.04	1.13–3.66	0.890	0.0057	1.85	1.10–3.13	0.889	0.0090	1.72	0.91–3.26	0.885	0.0558
Fragmentation (RR)	PIP	3.12	1.91–5.10	0.905	4.1E-7	2.29	1.49–3.52	0.897	6.0E-5	2.68	1.72–4.18	0.901	1.5E-6
	AISL	3.35	1.99–5.62	0.907	1.6E-7	2.33	1.50–3.62	0.897	4.5E-5	2.81	1.78–4.43	0.902	6.6E-7
	PSS	3.66	2.26–5.94	0.912	2.3E-9	2.49	1.62–3.81	0.900	7.4E-6	2.48	1.72–3.59	0.904	1.4E-7
	PAS	2.20	1.19–4.06	0.891	0.0032	1.98	1.16–3.38	0.890	0.0049	1.81	0.95–3.45	0.885	0.0399
Traditional short-termHRV	rMSSD	1.06	0.730–1.53	0.882	0.76	1.24	0.825–1.86	0.884	0.28	0.929	0.661–1.31	0.881	0.68
	pNN20	1.19	0.863–1.65	0.884	0.28	1.25	0.900–1.72	0.884	0.18	1.04	0.751–1.43	0.882	0.83
	pNN50	1.09	0.787–1.50	0.882	0.61	1.14	0.807–1.60	0.883	0.46	0.974	0.718–1.32	0.882	0.87
	SDSD	1.43	0.554–3.71	0.884	0.21	1.66	0.586–4.72	0.884	0.24	0.934	0.664–1.31	0.881	0.70
	HF	1.39	0.723–2.66	0.884	0.26	1.69	0.723–3.95	0.885	0.15	0.968	0.665–1.41	0.882	0.87
Non-linear	α_1_	0.454	0.292–0.707	0.897	1.39E-4	0.293	0.171–0.500	0.906	3.61E-7	0.810	0.566–1.16	0.884	0.24
	SampEn	1.16	0.845–1.58	0.883	0.36	1.57	1.14–2.17	0.890	0.0048	1.16	0.843–1.60	0.883	0.36

*The analysis was performed using raw measures. Values presented are the normalized odds ratio (OR_n_) and the 95% confidence intervals (95% CI) for the variables listed in the header column, in models adjusted for age and sex; the area under the receiver operating characteristic curve (AUC) and the p-value for the likelihood-ratio test of the null hypothesis that the addition of the HRV measure does not improve the fit of the model with age and sex alone. PIP, percentage of inflection points; IALS, inverse of the average length of the acceleration/deceleration segments; PPS, percentage of NN intervals in short segments; PAS, percentage of NN intervals in alternation segments. rMSSD, root mean square of the successive differences; pNN20 and pNN50, percentage of differences between successive NN intervals above 20 and 50 ms, respectively; SDSD, standard deviation of successive differences; HF, high frequency spectral power; α_1_, detrended fluctuation analysis short-term exponent; SampEn, sample entropy*.

Adding any of the fragmentation indices derived from NN interval time series to a model of CAD with the percentages of supraventricular premature beats (% SVPBs) significantly increased its performance (p < 0.00001) for all time periods. Specifically, while the AUC for the model with the % SVPBs was 0.754, the AUCs for the models that also included PIP, IALS, PPS, or PAS derived from NN intervals time series, were 0.852, 0.856, 0.866, and 0.771, respectively.

None of the associations between traditional time and frequency domain measures and CAD remained significant for any of the time periods when the models were adjusted for age and sex (Table 4). For the nonlinear measure α_1_, the associations remained significant during the 24-h and awake periods. For SampEn, the association with CAD during awake was significant, while the relationship during sleep time lost significance. The adjusted models with α_1_, for the 24-h and awake periods, and with SampEn, for the awake period, were superior to the model with only age and sex.

### Relationship between HRV measures and mean heart rate

**Figure 4:**
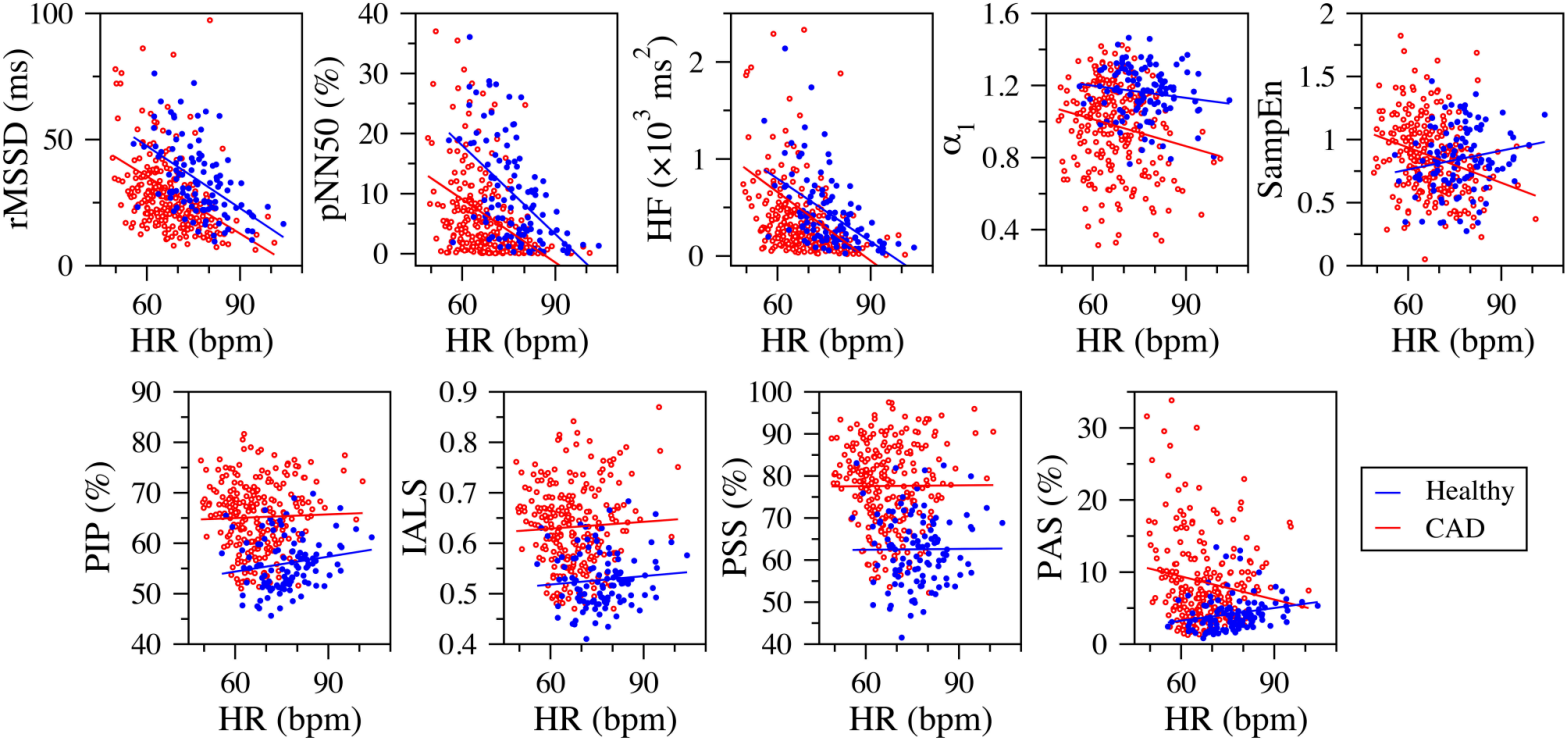
**Scatter plots of the traditional heart rate variability (rMSSD, pNN50, and HF), nonlinear (α_**1**_ and SampEn) and fragmentation (PIP, IALS, PPS, and PAS) indices vs. mean heart rate (in beats per minute, bpm) for the group of healthy subjects (blue dots) and those with coronary artery disease (CAD, red circles), derived from the analysis of 24-h NN interval time series**. The blue and red lines are the linear regression lines for the healthy and CAD groups, respectively. rMSSD, root mean square of the successive differences; pNN50, percentage of differences between successive NN intervals above 50 ms; HF, high frequency spectral power; α_1_, detrended fluctuation analysis short-term exponent; SampEn, sample entropy; PIP, percentage of inflection points; IALS, inverse of the average length of the acceleration/deceleration segments; PPS, percentage of NN intervals in short segments; PAS, percentage of NN intervals in alternation segments.

In both healthy subjects and those with CAD, the fragmentation indices, PIP, IASL, and PSS, were not significantly correlated with 24-h mean heart rate (Figure 4). The same was true for the group of patients with CAD. PAS was weakly correlated with mean heart rate in both healthy subjects and patients with CAD. The correlation was positive, *r* = 0.221 (*p* = 0.021), for the group of healthy subjects and negative, *r* = −0.146 (*p* = 0.020) in the group of those with CAD.

In contrast, the traditional time and frequency domain measures were strongly correlated with mean heart rate. The standardized Pearson correlation coefficients varied between 0.501 and 0.676 (*p* < 0.0001) in the group of healthy subjects and between 0.111 and 0.574 in the group of patients with CAD (*p* < 0.0001 for all correlations, but the one with SDSD).

SampEn was only weakly correlated with mean heart rate. The correlation was negative in the healthy group, *r* = −0.185, *p* = 0.054 and positive in the CAD group, *r* = 0.287, *p* < 0.0001. α_1_ was positively correlated with mean heart rate in patients with CAD *r* = 0.156, *p* < 0.013. For the healthy subjects, the correlation was not significant.

## Discussion

This study is of potential interest because it presents a new way of assessing short-term HRV under free-running (spontaneous) conditions. The novel methodology and findings are described under the rubric of sinus rate fragmentation (or conversely, smoothness or “fluency”). The conceptual framework is described in the Introduction. We found that the degree of fragmentation of the NN and RR time series, derived from 24-h Holter monitoring, varied directly as a function of cross-sectional age in a cohort of healthy young to elderly male and female subjects in sinus rhythm. In this group, older age was associated with increased fragmentation. This correlation was noted for the entire 24-h period, as well as, during putative wake and sleep periods. *Furthermore, we found that the fragmentation indices outperformed standard time and frequency domain measures, as well as, two widely used nonlinear measures (DFA* α_1_ *and SampEn), in separating healthy subjects from patients with CAD*.

In this study, we chose to analyze open access Holter data from groups of subjects whose clinical status was well-characterized and presented very sharp population differences: a group of healthy subjects and a group of patients with overt CAD. The healthy subjects were, on average, 20 years younger than the patients with CAD. Therefore, these two group were robustly separated by a model that simply incorporated age and gender (AUC = about 0.88). We expected the combination of older age and overt cardiovascular disease in the CAD group to enhance the ability of quantitative methods of HRV to unambiguously discriminate between patients and healthy individuals.

A potential link between aging and a variety of overt cardiovascular disease processes is the role of inflammation and fibrosis (Biernacka and Frangogiannis, 2011; Ghiassian et al., 2016), which decrease the amount and/or effectiveness of physiologic vagal tone modulation and promote the breakdown of regulatory networks, such as those controlling heart rate. Therefore, all short-term HRV measures were expected to change in the same directional way with aging and cardiovascular disease, for all time periods. In this regard, and in accord with “canonical” HRV precepts (HRV, 1996; Stauss, 2003; Billman, 2011), we hypothesized that traditional short-term HRV measures would decrease with cross-sectional age in the healthy group and that these measures would be lower for the patients with CAD than healthy subjects. In addition, we hypothesized that the fragmentation indices would increase with the participants' age in the healthy group and that they would be higher for patients with CAD than healthy subjects.

We found strong correlations between traditional HRV measures and the participants' age in the healthy group, for all time periods. These findings were in agreement with the generally accepted idea that HRV measure are useful to assess changes in heart rate dynamics with healthy aging (Pikkujamsa et al., 1999). In contrast, for the group of patients with CAD, traditional HRV indices, with only two exceptions, pNN20 and pNN50 during putative sleep, did not significantly change with the participants' age. This counterintuitive finding (Figure 2) may be due to the confounding effects of heart rate fragmentation, which may increase high-frequency variability not due to physiologic respiratory-vagal modulation (Domitrovich and Stein, 2002; Stein, 2002; Stein et al., 2005, 2008).

Furthermore, the ability of traditional short-term HRV measures to separate the healthy and CAD groups was also surprisingly poor. In models adjusted for age and sex, none of the traditional HRV measures significantly discriminated these two groups. Even in unadjusted models, the discriminatory power of conventional HRV measures was not consistent across time periods. In particular, HF power, traditionally interpreted as a measure of vagal tone modulation, did not discriminate the two groups for any of the time periods considered. Our results are consistent with previous cautionary reports about the utility of traditional HRV measures to assess vagal tone modulation especially with advanced age or underlying heart disease (Stein, 2002; Stein et al., 2005; Burr, 2007; Billman, 2013).

The nonlinear indices also did not provide consistent results. For example, α_1_ significantly increased with the participants' age during sleep, in both Pearson and Spearman correlation analyses. However, an inverse relationship was found for the awake and 24-h periods, in Pearson but not in Spearman analyses. In addition, lower α_1_ values were significantly associated the presence of CAD during the awake and 24-h periods, but not during sleep. The degree of randomness of heart rate time series, measured by SampEn, significantly decreased with the participants' age during the awake and sleep periods. Furthermore, while in an unadjusted model, a one standard deviation increase in the degree of randomness of heart rate time series was associated with a 36% decrease in the odds of CAD during sleep time, in a models adjusted for age and sex, a one standard deviation increase in SampEn was associated with a 57% increase in the odds of CAD during awake period. For the other time periods, the associations were not significant.

The inconsistencies of the two nonlinear methods, α_1_ and SampEn, are not entirely unexpected. For example, the fluctuation function, *F*(*n*), in DFA analysis of 24-h NN interval time series usually presents a crossover separating “short-term” from “longer-term” behavior. However, the scale at which that crossover occurs may vary substantially from subject to subject. In addition, the degree of linearity of *F*(*n*) also tends to vary from subject to subject. SampEn, on the other hand, can be affected by nonstationarities that are common in real world data. In addition, fragmented time series can be highly predictable (leading to low SampEn), as in the case of those with a high density of alternation, or highly irregular, as in the case of “erratic” sinus rhythm (leading to increased SampEn).

The limitations of traditional and newer HRV methods, exemplified by the results reported above and those described by other investigators (Stein, 2002; Stein et al., 2005; Burr, 2007; Billman, 2013), help motivate the on-going searches for alternative approaches. The introduction of the concept of fragmentation of heart rate dynamics, accompanied by a set of metrics for its quantification, are part of this exploration.

Speculatively, possible mechanisms of the observed fragmentation include the breakdown of one or more components of the regulatory network controlling heart rate dynamics. An obvious first question would be whether the higher fragmentation values in CAD vs. the healthy group could simply be due to SVPBs mislabeled as normal sinus beats. While the THEW website describes that three lead Holter monitor recordings were first processed using an automated beat annotation program and then subjected to visual review and adjudication, the possibility that some of the beats labeled as N are actually subtle SVPBs, and not sinus beats, cannot be absolutely excluded. To address this possible confounder, one would assume that the recordings with the highest likelihood of containing hidden SVPBs would be those with the highest percentage of labeled SVPBs. Therefore, to assess whether our results were likely a consequence of mislabeled SVPBs, we compared the performance of a model of CAD with the % SVPBs, as the sole independent variable to that of a model with the % SVPBs and each fragmentation index. We found that all fragmentation indices added significant information to the model with % SVPBs. This finding supports the contention that fragmentation is not a surrogate measure of “hidden” supraventricular ectopy.

A second question would be whether abnormalities in breathing dynamics could be responsible for the fragmentation of heart rate dynamics through respiratory-cardiac coupling. Since we did not have a direct measure of respiration, we cannot exclude the possibility that inter-breath interval time series were themselves fragmented. However, such a mechanism is unlikely since beat-to-beat changes in the sign of heart rate acceleration are above the frequency response of the vagal-sinus modulatory system. In fact, the coupling between the sinus node and the vagus tends to drop-off at high respiratory rates (Angelone and Coulter, 1964; Hirsch and Bishop, 1981). Furthermore, the most erratic variants of sinus rhythm are seen in the populations of older healthy subjects and those with organic heart disease (Stein et al., 2005, 2008), groups with the most impaired vagal modulatory capacity and, therefore, those least likely to show very high frequency coupling between autonomic and electrophysiologic components.

The specific electrophysiologic bases for fragmentation of heart rate dynamics remain to be determined. More than one mechanism may be contributory. For example, alternans phenotypes could be due to sinus node exit block or to very subtle atrial bigeminy with SVPBs originating near or even within the sino-atrial (SA) node (Geiger and Goerner, 1945). Another mechanism that could account for fragmentation would be modulated sinus node parasystole (Jalife et al., 1986), an arrhythmia in which two pacemaker sites in the SA area show bidirectional coupling and appear to “compete” for control of the heartbeat. Under certain parameter regimes, such coupling may induce a variety of alternating NN patterns (Jalife et al., 1986). The underlying electrophysiologic mechanisms to account for fragmentation may also involve perturbations of internal pacemaker “clocks” in the SA node (Lakatta et al., 2010). From a pathophysiologic viewpoint, mechanisms related to altered conduction and/or abnormal automaticity all reflect instabilities in the parasympathetic-SA node-atrial network. Given this substrate of instability, highly fragmented (whether erratic or periodic) types of NN patterns may represent pre- or even pro-arrhythmic markers. Our findings that fragmentation is increased in the elderly and in those with established CAD support this contention. Fragmentation would be of high interest if it were a forerunner of arrhythmias such as atrial fibrillation or other tachyarrhythmias in which the control network becomes so unstable that sinus node function is overridden by ectopic stimuli. Whether fragmentation is an independent risk marker of cardiovascular mortality related to heart failure also remains to be determined, as does any relationship to classical sick sinus syndrome.

More generally, the findings here support a modification in the standard classification of sinus rhythm into “phasic” and “non-phasic” variants (Faulkner, 1930; Hirsch and Bishop, 1981; Fisch and Knoebel, 2000). The first category refers to the oscillations in heart rate that are coherent with respiration and are most marked in younger individuals at rest, during deep sleep or with meditation (classic RSA). Non-phasic sinus arrhythmia, a term that has largely disappeared from the clinical lexicon, has been used to refer to a variety of sinus variants without this strict periodicity, including erratic sinus rhythm, and usually connotes abnormal sinus function (Stein et al., 2005). However, non-phasic types of sinus arrhythmia may also occur as physiologic variants, e.g., during exercise and recovery.

An alternative schema would be classify sinus rhythm into phasic and non-phasic types, and then sub-divide non-phasic into either physiologic due to short term trends but without tight respiratory coupling and non-physiologic, i.e., fragmented categories. However, we emphasize that fragmentation analysis *per se* does not separate phasic and non-phasic variants into two discrete bins. Rather, it quantifies, in a continuous way, the degree to which fragmentation is present.

Heart rate fragmentation may account for some of the abnormal patterns in Poincaré and other maps previously reported (Woo et al., 1992; Brouwer et al., 1996; Huikuri et al., 1996; Domitrovich and Stein, 2002; Stein et al., 2005, 2008; Gladuli et al., 2011; Makowiec et al., 2015). Such maps contain important information about the temporal structure of a time series. However, they are difficult to quantify in a physiologically interpretable way. Commonly employed metrics such as SD1, SD2, and SD1/SD2, only measure linear properties of the data that are also captured by time domain HRV measures such as rMSSD and SDSD (Brennan et al., 2001). If heart rate fragmentation is found to be one of the mechanisms underlying such abnormal patterns, the metrics introduced here may help identify, in a fully automated way, the time series associated with certain types of anomalous Poincaré plots.

Other fragmentation-related indices may also prove useful. Examples include the densities of: (i) “hard edges,” defined as inflection points for which Δ*NN*_*i*_ × Δ*NN*_*i*+1_ < 0; (ii) “soft edges,” defined as Δ*NN*_*i*_ × Δ*NN*_*i*+1_ = 0, where Δ*NN*_*i*_ ≠ Δ*NN*_*i*+1_; (iii) “short segments,” defined as acceleration/deceleration segments encapsulated between “hard edges” or “soft edges;” and (iv) segments for which heart rate does not change. Additionally, symbolic dynamical analysis of heart rate increment time series, where words are analyzed in terms of the number of edges they contain may also prove useful in this context.

Finally, the fragmentation indices have a number of attractive features. First, these indices are independent of the mean heart rate (Figure 4). The only exception is PAS, which is not a general fragmentation index, but quantifies a particular type of fragmentation (pattern of the type “ABAB,” where “A” and “B” represent increments of opposite sign). In contrast, traditional short-term time and frequency domain measures showed highly significant negative associations with mean heart rate, both in the group of healthy subjects and of those with CAD. These results are in line with those reported in other studies (Monfredi et al., 2014; Sacha, 2014). Second, by construction, the fragmentation indices (including PAS) are also independent of the amplitude of the time series. This feature is due to the fact that accelerations/decelerations were defined as increments/decrements in heart rate of any magnitude. Thus, two time series with fluctuation patterns that only differ in amplitude (e.g., time series, *u*_*i*_ and *v*_*i*_, for which *u*_*i*_/*v*_*i*_ = *c*, where *c* is a constant) will have exactly the same degree of fragmentation. Future studies will be needed to explore whether the use of a threshold >0 in the definition of accelerations and decelerations further increases the discriminatory power of these measures in HRV analyses. Third, the fragmentation indices are among the measures least affected by nonstationarities. The reason is that the operation of calculating the increment time series, used to detect the inflection points (i.e., changes in heart rate acceleration sign), detrends the data. Fourth, the fragmentation indices can be computed using NN or RR interval time series. Indeed the use of the latter did not impair the discriminatory power of the fragmentation indices for the populations studied here. This finding, if validated, may facilitate fully automated implementations of the method. Future studies will also help determine the effect of data length on the confidence intervals of the fragmentation indices.

## Conclusion

Analysis of short-term HRV is enhanced by a set of computational tools that quantify the fragmentation of heartbeat variability, defined by abrupt changes in the sign of HR acceleration. In a Holter monitor database from healthy subjects, the degree of fragmentation increased with the participants' age. Furthermore, fragmentation measures outperformed traditional short-term measures of HRV in discriminating a group of patients with CAD and from the healthy subjects. Fragmentation of sinus rhythm cadence may support a new class of dynamical biomarkers that probe the integrity of the regulatory network comprising neuroautonomic, sinus node and atrial components.

## Author contributions

MC and AG developed the fragmentation concept and method. RD directed the statistical analysis. All three authors contributed to the interpretation of the findings and worked collaboratively on the manuscript.

### Conflict of interest statement

The authors declare that the research was conducted in the absence of any commercial or financial relationships that could be construed as a potential conflict of interest.

## Abbreviations

α_1_: detrended fluctuation analysis short-term exponent
a.u.: Arbitrary units
AUC: Area under the receiver operating characteristic curve
CAD: Coronary artery disease
DFA: Detrended fluctuation analysis
ECG: Electrocardiogram
*F*(*n*): DFA root-mean-square fluctuation function of the integrated and detrended data, computed using windows of length n
HF: High frequency spectral power
HRV: Heart rate variability
IALS: Inverse of the average length of the acceleration/deceleration segments
IDEAL: Intercity Digital Electrocardiogram Alliance
NN: Normal-to-normal (sinus) interbeat interval
OR_n_: Normalized odds ratio
PAS: Percentage of NN intervals in alternation segments
PIP: Percentage of inflection points
pNN20: Percentage of differences between successive NN intervals above 20 ms
pNN50: Percentage of differences between successive NN intervals above 50 ms
PSS: Percentage of NN intervals in short segments
RR: Cardiac interbeat interval
RSA: Respiratory sinus arrhythmia
rMSSD: Root mean square of successive differences
SA: Sino-atrial node
SampEn: Sample entropy
SDSD: Standard deviation of successive differences
SVPB: Supra-ventricular premature beat
THEW: Telemetric and Holter ECG Warehouse.

## References

[B1] Angelone A. Coulter N. A. Jr. (1964). Respiratory sinus arrhythmia: a frequency dependent phenomenon. J. Appl. Physiol. 19, 479–482. 14173545 10.1152/jappl.1964.19.3.479

[B2] Bergfeldt L. Haga Y. (2003). Power spectral and Poincaré plot characteristics in sinus node dysfunction. J. Appl. Physiol. 94, 2217–2224. 10.1152/japplphysiol.01037.2002 12576413

[B3] Biernacka A. Frangogiannis N. G. (2011). Aging and cardiac fibrosis. Aging Dis. 2, 158–173. 21837283 PMC3153299

[B4] Billman G. E. (2011). Heart rate variability - a historical perspective. Front. Physiol. 2:86. 10.3389/fphys.2011.00086 22144961 PMC3225923

[B5] Billman G. E. (2013). The LF/HF ratio does not accurately measure cardiac sympatho-vagal balance. Front. Physiol. 4:26. 10.3389/fphys.2013.00026 23431279 PMC3576706

[B6] Binkley P. F. Eaton G. M. Nunziata E. Khot U. Cody R. J. (1995). Heart rate alternans. Ann. Intern. Med. 122, 115–117. 10.7326/0003-4819-122-2-199501150-00007 7503828

[B7] Brennan M. Palaniswami M. Kamen P. (2001). Do existing measures of Poincaré plot geometry reflect nonlinear features of heart rate variability? IEEE Trans. Biomed. Eng. 48, 1342–1347. 10.1109/10.959330 11686633

[B8] Brouwer J. van Veldhuisen D. J. Man in't Veld A. J. Haaksma J. Dijk W. A. Visser K. R.. (1996). Prognostic value of heart rate variability during long-term follow-up in patients with mild to moderate heart failure. The Dutch Ibopamine Multicenter Trial Study Group. J. Am. Coll. Cardiol. 28, 1183–1189. 10.1016/S0735-1097(96)00279-3 8890814

[B9] Burr R. L. (2007). Interpretation of normalized spectral heart rate variability indices in sleep research: a critical review. Sleep 30, 913–919. 17682663 10.1093/sleep/30.7.913 PMC1978375

[B10] Domitrovich P. P. Stein P. K. (2002). A new method to detect erratic sinus rhythm in RR-interval files generated from Holter recordings. Comput. Cardiol. 26, 665–668. 10.1109/CIC.2002.1166860

[B11] Faulkner J. M. (1930). The significance of sinus arrhythmia in old people. Am. J. Med. Sci. 180, 42–46. 10.1097/00000441-193007000-00006

[B12] Fisch C. Knoebel S. (2000). Electrocardiography of Clinical Arrhythmias. Armonk, NY: Future Publishing Company.

[B13] Friedman B. (1956). Alternation of cycle length in pulsus alternans. Am. Heart J. 51, 701–712. 10.1016/S0002-8703(56)80006-9 13302142

[B14] Geiger A. Goerner J. (1945). Premature beats of sinus origin: electrocardiographic demonstration of a clinical case. Am. Heart J. 30, 284–291. 10.1016/S0002-8703(45)90007-X

[B15] Ghiassian S. D. Menche J. Chasman D. I. Giulianini F. Wang R. Ricchiuto P.. (2016). Endophenotype network models: common core of complex diseases. Sci. Rep. 6:27414. 10.1038/srep27414 27278246 PMC4899691

[B16] Gladuli A. Moise N. S. Hemsley S. A. Otani N. F. (2011). Poincaré plots and tachograms reveal beat patterning in sick sinus syndrome with supraventricular tachycardia and varying AV nodal block. J. Vet. Cardiol. 13, 63–70. 10.1016/j.jvc.2010.12.001 21288788 PMC3296454

[B17] Goldberger A. L. Amaral L. A. N. Glass L. Hausdorff J. M. Ivanov P. C. Mark R. G.. (2000). PhysioBank, PhysioToolkit, and PhysioNet: components of a new research resource for complex physiologic signals. Circulation 101, e215–e220. 10.1161/01.CIR.101.23.e215 10851218

[B18] Hirsch J. A. Bishop B. (1981). Respiratory sinus arrhythmia in humans: how breathing pattern modulates heart rate. Am. J. Physiol. 241, H620–H629. 7315987 10.1152/ajpheart.1981.241.4.H620

[B19] Huikuri H. V. Seppanen T. Koistinen M. J. Airaksinen J. Ikaheimo M. J. Castellanos A.. (1996). Abnormalities in beat-to-beat dynamics of heart rate before the spontaneous onset of life-threatening ventricular tachyarrhythmias in patients with prior myocardial infarction. Circulation 93, 1836–1844. 10.1161/01.CIR.93.10.1836 8635263

[B20] HRV (1996). Heart rate variability: standards of measurement, physiological interpretation and clinical use. Task Force of the European Society of Cardiology and the North American Society of Pacing and Electrophysiology. Circulation 93, 1043–1065. 10.1161/01.CIR.93.5.1043 8598068

[B21] Jalife J. Michaels D. C. Langendorf R. (1986). Modulated parasystole originating in the sinoatrial node. Circulation 74, 945–954. 10.1161/01.CIR.74.5.945 2429784

[B22] Kuo T. B. Lin T. Yang C. C. Li C. L. Chen C. F. Chou P. (1999). Effect of aging on gender differences in neural control of heart rate. Am. J. Physiol. 277(6 Pt 2), H2233–H2239. 10600841 10.1152/ajpheart.1999.277.6.H2233

[B23] Lakatta E. G. Maltsev V. A. Vinogradova T. M. (2010). A coupled SYSTEM of intracellular Ca2+ clocks and surface membrane voltage clocks controls the timekeeping mechanism of the hearts pacemaker. Circ. Res. 106, 659–673. 10.1161/CIRCRESAHA.109.206078 20203315 PMC2837285

[B24] Lewis T. (1920). The Mechanism and Graphic Registration of the Heart Beat. New York, NY: PB Hoeber.

[B25] Makowiec D. Wejer D. Kaczkowska A. Żarczyńska-Buchowiecka M. Struzik Z. R. (2015). Chronographic imprint of age-induced alterations in heart rate dynamical organization. Front. Physiol. 6:201. 10.3389/fphys.2015.00201 26236241 PMC4501288

[B26] Mietus J. E. Peng C.-K. Henry I. Goldsmith R. L. Goldberger A. L. (2002). The pNNx files: re-examining a widely used heart rate variability measure. Heart 88, 378–380. 10.1136/heart.88.4.378 12231596 PMC1767394

[B27] Monfredi O. Lyashkov A. E. Johnsen A. B. Inada S. Schneider H. Wang R.. (2014). Biophysical characterization of the underappreciated and important relationship between heart rate variability and heart rate. Hypertension 64, 1334–1343. 10.1161/HYPERTENSIONAHA.114.03782 25225208 PMC4326239

[B28] Peng C.-K. Havlin S. Stanley H. E. Goldberger A. L. (1995). Quantification of scaling exponents and crossover phenomena in nonstationary heartbeat time series. Chaos 5, 82–87. 10.1063/1.166141 11538314

[B29] Pikkujamsa S. M. Makikallio T. H. Sourander L. B. Raiha I. J. Puukka P. Skytta J.. (1999). Cardiac interbeat interval dynamics from childhood to senescence. Circulation 100, 393–399. 10.1161/01.CIR.100.4.393 10421600

[B30] Richman J. S. Moorman J. R. (2000). Physiological time-series analysis using approximate entropy and sample entropy. Am. J. Physiol. Heart Circ. Physiol. 278, H2039–H2049. 10843903 10.1152/ajpheart.2000.278.6.H2039

[B31] Sacha J. (2014). Interaction between heart rate and heart rate variability. Ann. Noninvasive Electrocardiol. 19, 207–216. 10.1111/anec.12148 24602150 PMC6932101

[B32] Stauss H. M. (2003). Heart rate variability. Am. J. Physiol. Regul. Integr. Comp. Physiol. 285, R927–R931. 10.1152/ajpregu.00452.2003 14557228

[B33] Stein P. K. (2002). Heart rate variability is confounded by the presence of erratic sinus rhythm. Comput. Cardiol. 26, 669–672. 10.1109/CIC.2002.1166861

[B34] Stein P. K. Domitrovich P. P. Hui N. Rautaharju P. Gottdiener J. (2005). Sometimes higher heart rate variability is not better heart rate variability: results of graphical and nonlinear analyses. J. Cardiovasc. Electrophysiol. 16, 954–959. 10.1111/j.1540-8167.2005.40788.x 16174015

[B35] Stein P. K. Le Q. Domitrovich P. P. (2008). Development of more erratic heart rate patterns is associated with mortality post-myocardial infarction. J. Electrocardiol. 41, 110–115. 10.1016/j.jelectrocard.2007.11.005 18328334 PMC2323590

[B36] Thayer J. F. Yamamoto S. S. Brosschot J. F. (2010). The relationship of autonomic imbalance, heart rate variability and cardiovascular disease risk factors. Int. J. Cardiol. 141, 122–131. 10.1016/j.ijcard.2009.09.543 19910061

[B37] Woo M. A. Stevenson W. G. Moser D. K. Trelease R. B. Harper R. M. (1992). Patterns of beat-to-beat heart rate variability in advanced heart failure. Am. Heart J. 123, 704–710. 10.1016/0002-8703(92)90510-3 1539521

